# Human papilloma virus genotypes in dysplasia and epithelial hyperplasia of oral cavity using the luminex xmap technology. A multicenter study

**DOI:** 10.4317/medoral.23188

**Published:** 2019-12-24

**Authors:** Sandra Janeth Perdomo-Lara, María Rosa Buenahora, Efraín Álvarez, Farith González-Martínez, Martha Rebolledo, Fabio Ancisar Aristizabal, Carlos Humberto Colegial, Alvaro Horta, Jairo Bustillo, David Díaz-Báez, Carlos Martín Ardila, Gloria Inés Lafaurie

**Affiliations:** 1 Unit of Basic Oral Investigation-UIBO, School of dentistry, Universidad El Bosque, Bogotá-Colombia; 2 Cellular and Molecular Immunology Group-INMUBO, School of Dentistry, Universidad El Bosque, Bogotá-Colombia; 3 Universidad El Bosque, Unit of Oral Epidemiology Investigation-UNIECLO, School of dentistry, Bogotá-Colombia; 4 Biomedical Stomatology Research Group, Universidad de Antioquia U de A, Medellín-Colombia; 5 Public Health Investigation Group-GISPOUC, School of dentistry, Universidad de Cartagena. Cartagena. Colombia; 6 Dentistry Research Group-GIOUMEB, Universidad Metropolitana, Barranquilla-Colombia; 7 Institute of Biotechnology, Universidad Nacional de Colombia. Bogotá-Colombia; 8 Bio-Molecular Laboratory. Bogotá-Colombia

## Abstract

**Background:**

Oral cancer associated with high risk (HPV-HR) human papilloma virus (HPV) has been increasing. HPV-HR has been associated with epithelial dysplasia, however, little information exists on its frequency in epithelial hyperplasia lesions. The aim of this study is to compare HPV genotypes in dysplastic and hyperplastic lesions of oral cavity.

**Material and Methods:**

Two hundred and fifty oral lesions: 131 dysplasia and 119 hyperplasia from two regions of Colombia were evaluated. One hundred seventy-four coming from urban area and 104 from a high risk population to oral cancer from a rural area. HPV was identified by qPCR and Twenty-four HPVs genotypes were evaluated by Luminex® technology. Logistic regressions were performed to establish the associations between HPV infections with oral dysplasia.

**Results:**

Twenty-eight percent (70/250) of the samples were positives for any HPV and HPV-HRs were more frequently than low risk HPVs. HPV-16 was the most detected genotype (16%) followed by HPV-31, 53, 18 and 45. HPV, HPV-HRs and HPV-16 were only associated with dysplasia in urban area; OR 3.28 (CI 95% 1.49-7.17), OR 7.94 (CI 95% 2.97-21.2) and OR 5.90 (CI 95% 2.05-17). Individuals in rural area showed more HPV and HPV-HRs infection in hyperplasic lesions than urban population. The majority of HPV+ lesions had multi-type of HPV (52/70) and the urban individuals showed more genotypes than rural population.

**Conclusions:**

HPV-.HRs are frequently found in hyperplastic and dysplastic epithelial lesions. HPV-HRs and HPV-16 were associated with dysplasia in urban population. Rural high risk population and urban population differ in the frequency and variety of HPV genotypes.

** Key words:**Human papilloma virus, epithelial dysplasia, epithelial hyperplasia, HPV-genotypes.

## Introduction

While the incidence of cervical cancer has been decreasing in industrialized countries, the incidence of HPV in oral cavity and oropharynx cancers has been increasing over the past 20 years (1,2). An increase of the cases of cancers associated to HPV has been reported and a sustained increase of new cases of oropharynx squamous cell carcinoma (OPSCC) and oral squamous cell carcinoma (OSCC) will be associated with HPV in the future (3,4). However, the association between HPV and oropharyngeal cancer is much stronger than for oral cancer (5) and the association of HPV with OSCC is weak and it is still controversial (6).

HPV infection is considered a sexually transmitted disease, and some sexual practices as lifetime coital sex partnership numbers and the number of oral sex partners has been associated with HPV transmission to the oral mucosa (7-9) and may increase the risk of HPV-oral infections and generate important changes in the oral epithelium that increase the risk of carcinogenesis (10). The viral oncogenes of HPV inactivate two crucial human tumor suppressor genes: p53 (E6) and retinoblastoma protein (pRb) (E7). The inactivation of these genes results in loss of cell cycle control, altered cell differentiation, increased mutations, and chromosomal instability (11,12).

The working group of WHO in 2005 recommended the use of the term “oral potentially malignant disorder” (OPMD). These lesions refer to an altered epithelium with a higher probability of progression to squamous cell carcinoma and these changes involve epithelial dysplasia (13). However, in 2011, Sarode *et al*., (14) proposed a novel pathogenesis based classification of OPMDs in which all the disorders either carry some risk and predispose the oral mucosa to OSCC transformation and it included lesions such oral leukoplakia and conditions as oral lichen planus associated with HPV infection (15,16).

Based on their potential oncogenic activity, HPV subtypes have been divided into high-risk (HPV-HRs) and low risk (HPV-LRs) viruses. HPV-HR are associated with the development of cancer and are termed oncogenic viral types (17,18). HPV-HRs 16/18 genotypes have been associated with OPSCC, oropharyngeal dysplasia (OOPD), OSCC and OPMD-based dysplasia and enough evidence supports this association (19,20). OPMD-based dysplasia has been compared with normal mucosa but the comparison of HPV prevalence in epithelial dysplasia with epithelial hyperplasia is limited. HPV-HR having a great affinity for epithelial tissue and can penetrate and stay dormant in inflamed tissues such as periodontal tissue and can be detected from benign oral squamous epithelial lesions (21,22).

More of 100 different genotypes of HPV has been recognized and 15 have been classified as HPV-HRs (23,24). Recently, a new technologies for multiple detection of HPV genotypes has been development. A new technology quantifies simultaneously multiples genotypes using fluorescent beads, and gene-specific multiplex polymerase chain reaction and has been validated for use in clinical samples (25).

The purpose of this study is to identify the main genotypes of HPV in oral mucosal lesions according to histological changes by comparing lesions with OPMD-based dysplasia with those showing hyperplastic changes without dysplastic.

Material and methods

- Type of study

A cross sectional study was realized comparing the HPV genotypes in oral lesions with dysplasia and epithelial hyperplasic histologic diagnosis.

- Population

Two hundred and fifty oral mucosal lesions of individuals diagnosed in different services associated with the universities participants of two geographic regions of Colombia (Bogotá and the Caribbean region) were evaluated. One hundred thirtyone [131] lesions with dysplasia and 119 lesions with histologic diagnosis of hyperplasia without dysplasia were analyzed to identify 24 HPV genotypes by the Luminex® technique. One hundred twenty-three samples come from Bogota and 127 come from Caribbean Region. One hundred forty-six [146] individuals were evaluated from urban areas of the two geographic regions in retrospective and prospective evaluation and the samples were obtained from 2010 to 2018. A subset of samples [104] obtained in a specific high risk to oral cancer population associated with the habit of reverse smoking in rural area of Caribbean region (Sucre-Department) obtained in 2004 (26) also were analyzed.

The study was approved by the Ethics Committee of the School of Dentistry of Universidad El Bosque (Act. No. 012-2013). All samples evaluated were obtained with informed consent.

- Clinical and socio-demographic data

Socio-demographic data such as age (≤ 45 age/ >45 years), gender (male/female), region (Bogotá/Caribbean), area (rural/urban), date of diagnosis (≤ 5 years/ 5-10years/ >10 years) were obtained in all samples. Other data such as cigarette and alcohol consumption could not be obtained for all samples because many were obtained from seizures of oral pathology.

- Selection and exclusion criteria

Clinical diagnoses: Oral lesions not associated with oral infections (caries or periodontal disease) were included in this study. Lesions from different locations of the oral cavity were included, except for lesions of the oropharynx and soft palate or lesions of floor of the mouth including floor of the tongue. White, red, pigmented and lesions without color change such macules, papules, plaques, nodules, clinical hyperplasia, ulcers, erosions and blisters, were included for histological analysis. The lesions were included if in the histopathological analysis they showed a diagnosis of hyperplasia or epithelial dysplasia.

Histologic diagnoses: expert pathologists evaluated all samples in each region. The samples were classified into 1) with the presence of dysplasia 2) with the presence of epithelial hyperplasia without dysplasia. In dysplasia, the cells look abnormal: there are cells of differing sizes, misshapen cells, intensely pigmented cells and an uncommon number of cells presently dividing, but they are not cancer. In hyperplasia, there is an increase in the number of cells in an organ or tissue that appear normal under a microscope. The basal and para basal hyperplasia were excluded.

- DNA isolation from paraffin embedded tissue 

To minimize the potential for PCR contamination, the histotechnologists were instructed to wear gloves when handling blocks and to clean the microtome and the area around the cutting station before beginning work, and to use a new disposable microtome blade for each block. Six serial 5-μm sections were cut from each block. The sections were transferred directly from the microtome blade into a 1,5mL tube, yielding two tubes per block. Total DNA was extracted from the replicate tubes using the QI*Aa*mp® DNA FFPE Tissue kit (Qiagen, Valencia, CA). Briefly, the paraffin was removed by vortexing and 10 minutes incubation with 1.2 mL xylene, followed by two washes with pure (200 proof) ethanol. The air-dried pellet was then incubated with 20 μL proteinase K and 180 μL ATL lysis buffer for 16 hours in a heat block at 56°C. The lysed emulsion was further purified with the DNeasy spin-column kit. DNA was finally recovered in a single elution step with 100 μL AE solution from the kit.

- Quality Assessment of DNA and HPV identification

Quality of DNA was confirmed by PCR with primers PC03/PC04, which amplify a 110 bp fragment of the ß-globin gene. The PCR products were visualized by gel electrophoresis on 1% agarose gels stained with SYBR Safe to identify the amplified 110pb band. qRT-PCR was performed for detection of HPV in samples using GP5+/GP6+ consensus primers with Kappa SYBR Green mastermix kit (Kapa Biosystems®) and CFX96 Bio-Rad real-time thermal cycler (Biorad, Hercules, CA). Briefly, the amplification was performed in 25 μl reaction mix containing 0,2µM each of GP5+ and GP6+ primers and 30-50ng of genomic DNA in 5 ul of test volume. The amplification ramp included first step for 3 min at 95°C for initial denaturation, followed by denaturation cycle of 20 secs at 95°C, an annealing cycle of 15 secs at 48°C and an elongation and readout cycle of 30 secs at 72°C for 45 cycles. Genomic DNA from HPV positive cells lines CaSki, SiHa and HeLa was used as positive controls. The specificity of PCR product was verified by a dissociation curve analysis.

- Multiplex HPV genotyping with Luminex® Technology

The sample DNA extracted from paraffin embedded tissue was subjected to PCR amplification, using sets of biotinylated BGP5+/BGP6+ primers contained in the kit. Additionally, a pair of primers for the amplification of a ß-globin gene fragment were added to the PCR as a control. PCR products were added to the bead mix containing 26 distinct bead populations coupled to 24 HPV, one ß-globin and one Hybridization Control specific oligonucleotide probe. The ß-globin control serves as quality control for genomic DNA in the PCR. After thermal denaturation and hybridization of target sequences to the bead-bound probes, labeling of the hybridized biotinylated PCR products was achieved by R-Phycoerythrin labeled Streptavidin (reporter fluorescence). The beads were analyzed in a Luminex 100 reader (Luminex Corp.), which uses two lasers, one that recognizes the bead set by the internal bead color and another to quantify the reporter fluorescence on the bead. Based on the detection of specific HPV and ß-globin PCR products and their combination, the following evaluation scheme should be applied (provided that usual signal values for the Hybrididization control of > 200. For each run and each HPV type a background and cut-off value wa scalculated based on the signal of the negative control. The kit has a sensitivity of 100% (76/76) and specificity of 78.3% (624/797). The results were expressed in median fluorescence intensities (MIF) of ≥100 beads per sample.

- Statistical analysis 

Descriptive analyses of frequency distribution were carried out for all variables. Bivariate analysis was performed by chi-square/Fisher tests to establish associations of the HPV, HPV-HRs, HPV-16, and HPV-18 with oral dysplasia compared with hyperplasia. All variables of HPV infection which showed a significance level <0.10 in the bivariate analysis were included in a logistical regression analysis to establish the OR with confidence intervals of 95% adjusted to age and gender (Model 1) or adjusted to age, gender, region and area (rural/urban) (Model 2). For all logistic models the goodness of fit tests for logistic regression (Hosmer-Lemeshow test) were performed. The models were compared with using the likelihood ratio chi-square and the Bayesian information criterion (BIC). A multiple correspondence analysis was performed to establish the correlation between the different genotypes of HPV in multiple infections. All data analysis was undertaken using Stata (Intercooled v11.0; Stata Corp LP, College Station, TX).

## Results

- Characteristics of the samples

The samples were mostly lip, palate and oral mucosa and with less frequency of tongue and gingiva. One hundred and thirty-one had a histological diagnosis of dysplasia and 119 were lesions with hyperplasia. The lesions most frequently evaluated for both types of histological diagnosis were white lesions, although these were more frequent in lesions with dysplasia (54.2% vs. 32.7%) and most were plaque lesions. The red lesions were also frequent but the pigmented lesions were the least frequent. Leukoplakia was the most frequent diagnosis in both histopathological diagnosis but this was more frequent in dysplasia, however, only half of the lesions had a presumptive clinical diagnosis (Table 1).

**Table 1:**
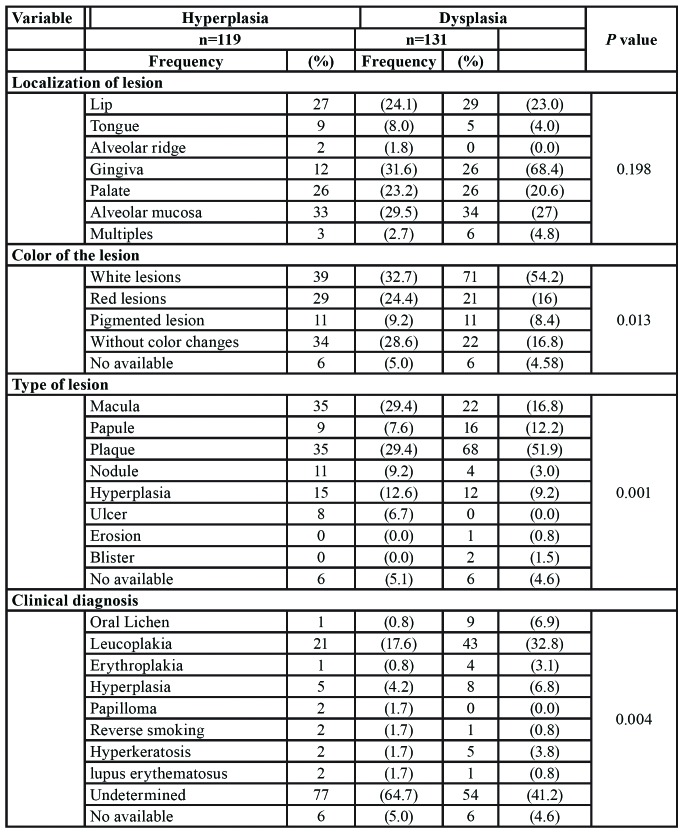
Clinical characteristics of the oral lesions.

- Characteristic of population

The socio demographic and clinic characteristics of the lesions evaluated are shown in Table 2. The majority of patients were older than 45 years old and women. A similar number of samples were evaluated for the two regions, although the samples were more from the urban area.

- HPV Frequency

70/250 (28%) of the samples were positives for any HPV evaluate by qPCR. The samples HPV+ were most frequently in samples with dysplasia than hyperplasia (32.8 vs. 22.7%) but the differences were not significant (*p*=0.75). HPV-HRs, HPV-16 and HPV-18 were also more frequently identified in dysplasia lesions (Table 2). HPV, HPV-HRs, HVP-16 and HPV-18 did not show significant associations with age nor by gender (female/male) (HPV; 27% vs., 29%; HPV-16; 17% vs., 16%) (*p*>0.05). (Data not shown).

**Table 2:**
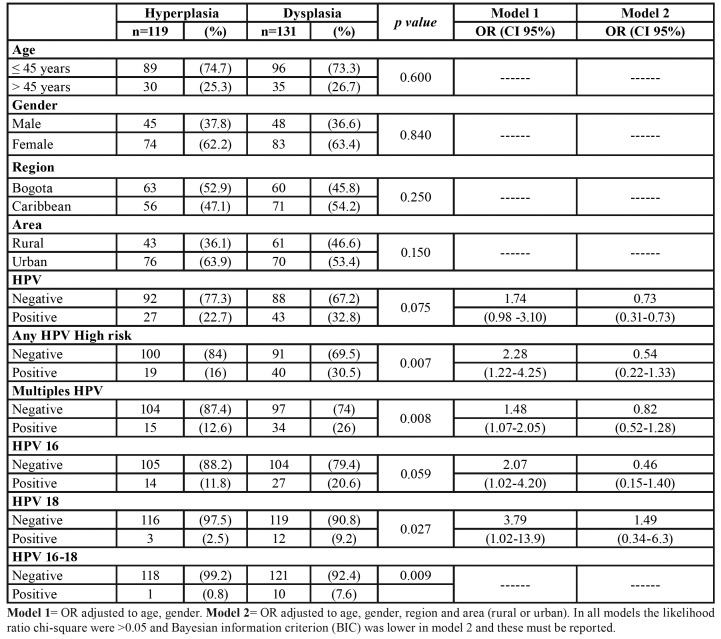
Association of socio-demographic and VPH with dysplastic lesions.

- HPV Genotypes

**Figure 1:**
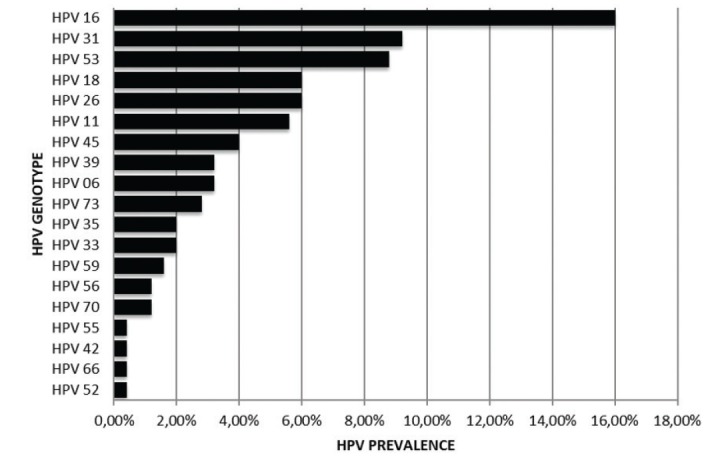
HPV genotypes in dysplastic and hyperplasic lesions.

In the majority of the lesions HPV+ analyzed, high-risk genotypes were detected. HPV-HRs were more frequently than HPV-LRs. HPV-16 was the most detected genotype in all samples and represent 16% of all samples analyzed. Other HPV-HRs with an important frequency were HPV-31, 18 and 45. HPV-53 showed also an important frequency but this is not classified in HPV-HRs group and has been considered a moderate risk. HPV-11 was the most frequently HPV-LRs detected (Fig. 1).

**Figure 2:**
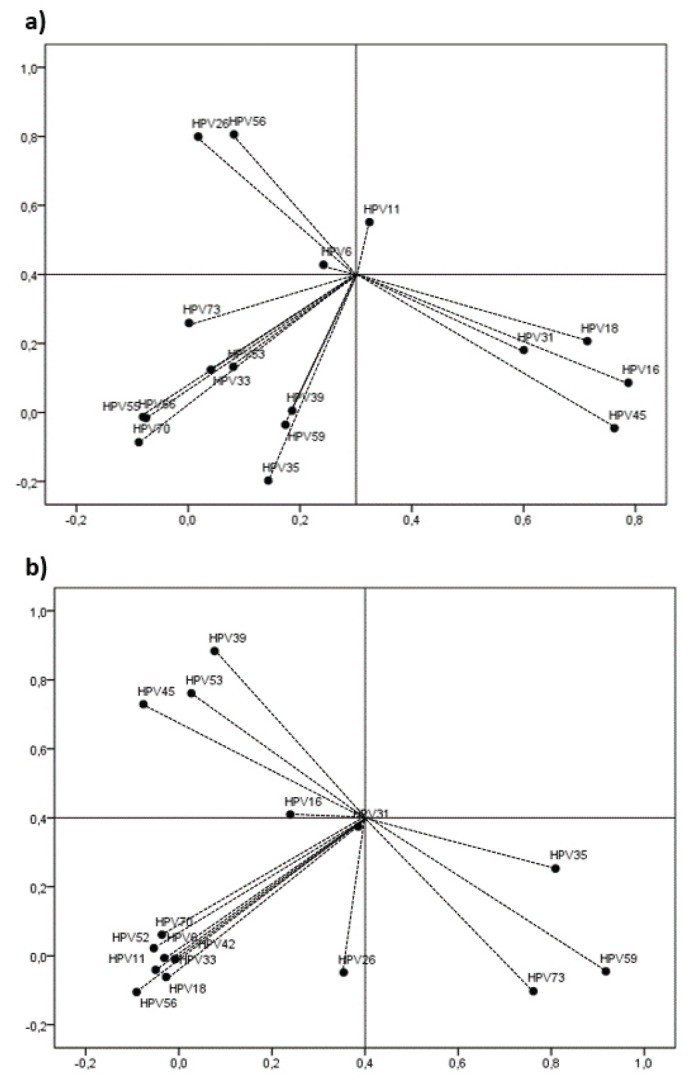
A multiple correspondence analysis of HPV genotypes in dysplasia an epithelial hyperplasia. a) Dysplasia, b) Hyperplasia.

Of the all positives genotypes detected, 59/250 (23.6%) were HPV-HRs +. HPV-16 was detected in 41/250 (16.4%), HPV-31 in 21/250 (8.4%), HPV-18, in 15/250 (6%), HPV-45 in 9/250 (3.6%), and HPV-33, HPV-35 and HPV-59, in less than 3% of the HPV+ samples. The HPV-LRs more frequently were HPV-11 in 9/250 (3.6%) and HPV-6 in 8/250 (3.2%). In only three cases the individuals were infected with one genotype, in the in the majority were multiple infections with other HPV-HRs viruses. Fifty-two 52/250 (20.8%) lesions showed infections by multiple HPV genotypes. Twenty-two/250 (8.8%) had infection by two genotypes (4.2% for hyperplasia vs. 12.98% for dysplasia) followed by 5 or more genotypes 11/250 (2.52% for hyperplasia vs. 6.11% for dysplasia). A lower frequency was observed for infections by 3 and 4 genotypes simultaneously (3.2 for hyperplasia vs. 4% for dysplasia). A multiple correspondence analysis is observed in Fig. 2. HPV 16 showed a high co-infection with HPV-HR such as HPV18, 31 and 45 in samples with dysplasia. In samples with hyperplasia, HPV showed co-infection with other HPV-HR as HPV 31, 39 and 53 but also with HPV 45 similar to dysplasia samples.

- HPV Genotypes in samples with dysplasia and hyperplasia

**Figure 3:**
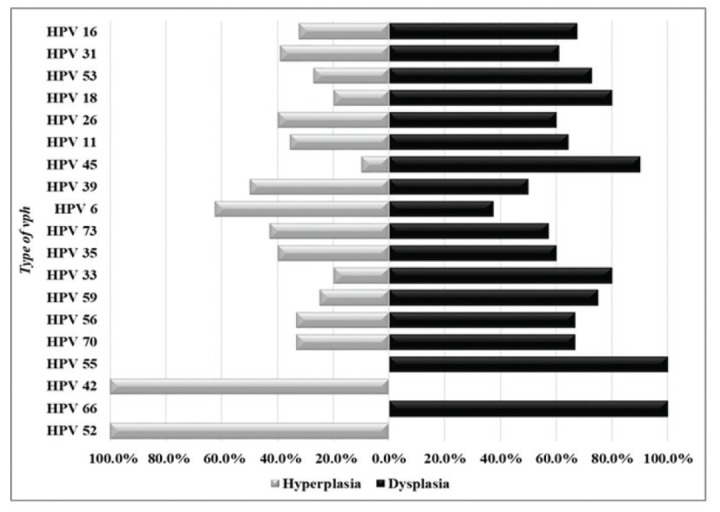
Comparison of HPV genotypes in dysplasia and hyperplasia lesions.

HPVs-HR were more frequently in dysplastic samples although they were also detected in hyperplasia. HPV-55 and HPV-66 were exclusively detected in samples of dysplasia, HPV-42 y HPV-52 only were detected in hyperplasia. The HPV-LRs more frequently in hyperplasia were HPV-6, HPV-11 (Fig. 3).

- Association of VPH with dysplastic lesions

Table 2 shows the associations between socio-demographic factors and the presence of HPV and HPV-HRs with lesions of dysplasia compared with lesions of hyperplasia. Age, gender, region and area did not show significant associations with the presence of dysplasia. HPV-HRs and multiple HPV infections showed significant associations with dysplasia adjusted to age and gender; OR 2.28 (CI 95% 1.22-4.25) and OR 1.48 (CI 95% 1.07-2.05) respectively. HPV 16, OR 2.07 (CI 95% 1.02 – 4.20) and HPV 18, OR 3.79 (CI 95% 1.02-13.9), were also associated with dysplasia. However, when adjusting by area and region, area (rural vs. urban) was a potential confounder variable and these associations were lost (Table 2).

In Table 3 is observed a stratified analysis for HPV infections for urban and rural samples. HPV presence was associated with dysplasia only in urban samples OR 3.28 (CI 95% 1.49-7.17). HPV-HR also was associated in urban samples OR 7.94 (CI 95% 2.97-21.2) and it was not significant for rural area OR 0.52 (CI 95% 0.21 – 1.29). HPV-16 was associated only in urban samples OR 5.90 (CI 95% 2.05-17) but HPV-18, HPV 18-16 and multiples infections showed a low frequency in the strata and it was not possible to find an accurate association with dysplasia in urban or rural samples (Table 3).

- HPV Genotypes in samples with dysplasia and hyperplasia in rural and urban samples

**Figure 4:**
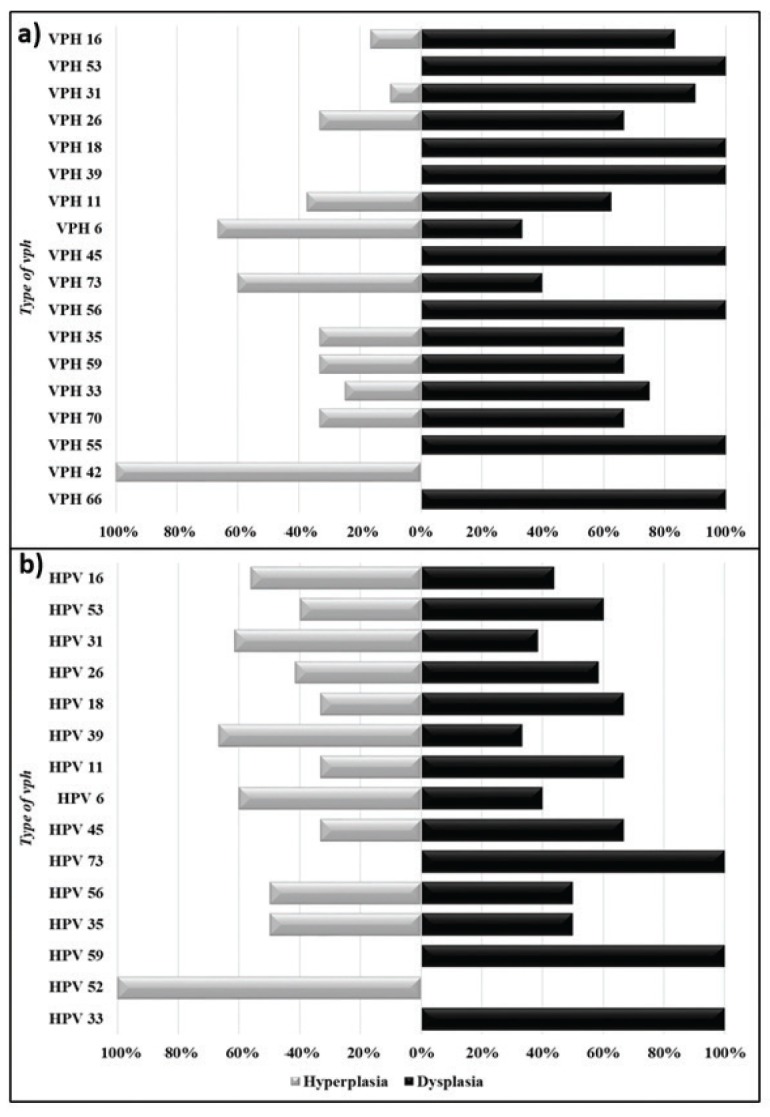
Comparison of HPV genotypes in rural and urban samples in hyperplasia and oral dysplasia. a) Genotypes in urban samples, b) Genotypes in rural samples.

Rural population showed more HPV-HRs genotypes in hyperplasic samples than urban samples (Fig. 3); HPV16, HPV18, HPV 31 showed higher frequencies in these samples (Fig. 3). In dysplasia samples urban showed more HPV-HRs genotypes than rural samples especially for HPV16, HPV18, HPV 31 and HPV45 (Fig. 3). Some genotypes such as HPV-42, 55, 66 and HPV-70 were identified exclusively in samples from urban areas and only HPV-52 was detected in rural samples (Fig. 4).

**Table 3:**
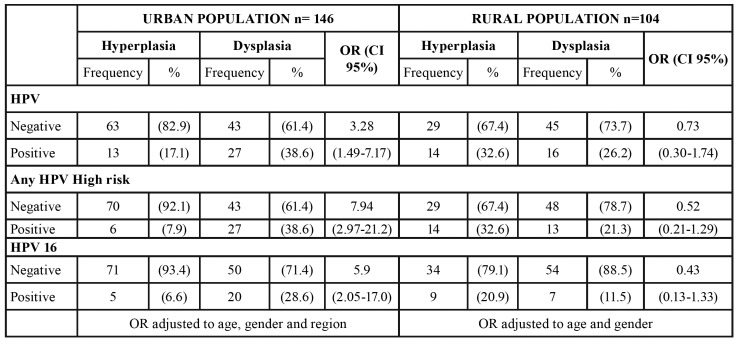
Association of HPV, HPV-HR, and HPV-16 with dysplastic lesions in a stratified analysis by urban or rural population.

## Discussion

The impact of HPV has been investigated in oral cancer in the last decades and the association with OPSCC, and OSCC has been well established (17,18). The longitudinal studies have not yet shown the predictive value of HPV-HR+ in development of oral cancer but a poor clearance of E7 antibody associated to HPV-16 are associated to OPSCC and OSCC recurrence (27,28). As an increase in oral cancer associated with HPV has been observed, the study of HPV infection in early stages has been a focus of attention in recent years (29,30). However, The HPV association is much stronger in tumors of the oropharynx than in oral cancer and the impact of HPV in oral cancer is still weak (6,31).

Different studies have suggested that HPV infection is probably a potential etiological factor of OPMD (32). The term OPMD included an altered epithelium with epithelial dysplasia (13). Based on these epithelial changes, this study was based on a histological diagnosis and not on a clinical diagnosis. Clinical diagnoses were lost in retrospective samples and in prospective samples some clinical diagnoses such as lichen, leukoplakia, erythroplakia and papilloma were included. However, other diagnoses but such as hyperplasia and hyperkeratosis were also included but do not correspond to a clinical diagnosis.

In this study, although the presence of high-risk genotypes was more frequent in lesions with dysplasia in urban population, these differences were not observed in the rural samples when these were compared with lesions with hyperplasia. HPV prevalence varies according to different studies and these differences seem to be related with sample size, detection methods, geographic populations, disease types, and HPV genotypes and it could be related with the type of control group used (32).

The prevalence of HPV in cancer-free individuals is low and has been reported in 7.7%; healthy patients had 1.4% of HPV-16 (33). However, in this study individuals with oral hyperplasia showed a prevalence of 22.7% of HPV-HRs. Oral hyperplasia is a common benign lesion of oral mucosa associated to different factors as trauma, medications or chronic irritation. Benning lesions such to multifocal epithelial hyperplasia, oral papilloma, vulgaris oral verruca and condyloma acuminatum are associated to HPV-LR such HPV-6, HPV-11 and HPV-13, but the presence of HPV-16 has been reported in a low frequency (22). The finding of a significant prevalence of HPV-HRs in epithelial hyperplasia could indicate that HPV-HRs may be in inflammatory lesions acting as reservoirs similar to inflamed gingival pocket epithelium which has been proposed as one of the reservoirs of HPV infection (21). However, it is also possible that lesions with epithelial hyperplasia are initial lesions of HPV-HRs infections and that the persistence of HPV can lead to a premalignant lesion.

In rural area the frequency of HPV-HRs was much higher than the one observed in urban areas in epithelial hyperplasia. In rural population exist significant disparities in knowledge and awareness of HPV and the HPV vaccine compared to urban (34). In our rural study, it is possible that less information about HPV infection has influenced a greater infection of hyperplasic lesions who did not show significant differences with dysplasia lesions. These rural samples evaluated in this study were included because they belonged to one of the participating centers and were obtained from a rural area of the Caribbean region with characteristics that differ from those obtained from the urban area. This population is a high-risk population for oral cancer associated with the habit of reverse smoking or artisanal tobacco (26) and it is possible that this factor combined with the sexual behaviors of these population are associated with increased HPV infection in oral tissues with inflammation.

The importance of HPV infection in oral epithelia in incipient lesions has focused on oral dysplasia. HPV-HR+ have been associated with dysplastic lesions when compared to normal mucous membranes (19). However, few studies have evaluated multiple genotypes of HPV in these oral mucosal lesions. An important finding in this study was that the majority of HPV-positive individuals showed multiple genotypes. Although HPV-16 was the most frequent genotype, the majority of the patients had multi-types associated. Data from 2009-2012 nationally representative sample of United States evaluated 37 HPV genotypes and the prevalence of multi-type (2-6 types) oral HPV infection was and 19.7% (22.0% for men, 12.1% for women) in those who had any type of oral HPV positivity. In contrast, in this study infection with multi-type of HPV was only slightly higher in men than in women (23% vs. 19%). The risk factors for multi-type HPV oral infection were current cigarette smoker and having a new sex partner in the past year (35). In this study the prevalence of multi-types was 13% in hyperplasia and 27% in dysplasia, but it was not possible to evaluate these factors in all individuals since many samples were obtained in pathology banks and did not have this information.

HPV-16 was the most important genotype of HPV in hyperplasic and dysplastic samples. HPV-16 has been the genotype most associated with OPSCC, OOPD, OSCC and OPMD followed by HPV-18 (19). In this study HPV-18 was not as frequent in the samples analyzed and another HPV-HR as HPV-31 was more prevalent. In Iranian population HPV-31 has the second genotype in frequency after HPV-18 in a study of multiple genotypes in samples of oral tumors and HPV-16 had a low frequency (36). HPV infection seems to be related to the geographical regions and a difference in frequency and genotypes were observed in rural and urban areas. Although the HPV-16 is still the HPV genotype most associated with OPSCC, the evidence is not yet conclusive to OSCC by the differences observed in different regions (37). Several studies showed that HPV DNA is present in a considerable number of OSCC, however, the presence of HPV-DNA does not mean the presence of a biologically active HPV per se. Further studies should be aimed at evaluating markers of viral activity by means of E6/E7 and cellular p16INK4A expression combined with survival studies (6).

The findings of this study do not support an association of HPV with oral epithelial dysplasia compared with epithelial hyperplasia because the results vary between geographical areas of the same country. The clinical significance of high-risk HPV loads in the malignant progression of oral potentially malignant disorders needs to be clarified. Recently, a retrospective cohort of premalignant oral lesions such oral lichen planus and dysplasia in Spain population evaluating HPV-DNA and p16INK4A expression followed by several years did not progress to invasive cancer (38). More studies with large population samples are required to confirm these findings.

## Conclusions

HPV, HPV-HRs and HPV-16 were more frequent in dysplasia lesions only in urban areas. HPV-16 is the most frequently genotype in the lesions evaluated but was associated with multi-type infection. HPV genotypes varies in rural and urban areas.

- Limitations

One limitation of this study was the lack of healthy oral tissue controls by ethics considerations. It was also not possible to obtain comparable samples from the population studied to obtain cytological samples to evaluate HPV. This study only compares hyperplasia and oral dysplasia samples. In this study it was not possible to establish associations of HPV with smoking habit because the information was not available. Other strong limitation of the study was the lack of clinical diagnosis in a great number of the cases.
